# Non-local crime density estimation incorporating housing information

**DOI:** 10.1098/rsta.2013.0403

**Published:** 2014-11-13

**Authors:** J. T. Woodworth, G. O. Mohler, A. L. Bertozzi, P. J. Brantingham

**Affiliations:** 1 Department of Mathematics, University of California, Los Angeles, CA 90095, USA; 2 Department of Mathematics and Computer Science, Santa Clara University, Santa Clara, CA 95053-0290, USA; 3 Department of Anthropology, University of California, Los Angeles, CA 90095, USA

**Keywords:** crime hotspots, density estimation, graph Laplacian, maximum penalized likelihood estimation, non-local means, Nyström's extension

## Abstract

Given a discrete sample of event locations, we wish to produce a probability density that models the relative probability of events occurring in a spatial domain. Standard density estimation techniques do not incorporate priors informed by spatial data. Such methods can result in assigning significant positive probability to locations where events cannot realistically occur. In particular, when modelling residential burglaries, standard density estimation can predict residential burglaries occurring where there are no residences. Incorporating the spatial data can inform the valid region for the density. When modelling very few events, additional priors can help to correctly fill in the gaps. Learning and enforcing correlation between spatial data and event data can yield better estimates from fewer events. We propose a non-local version of maximum penalized likelihood estimation based on the *H*^1^ Sobolev seminorm regularizer that computes non-local weights from spatial data to obtain more spatially accurate density estimates. We evaluate this method in application to a residential burglary dataset from San Fernando Valley with the non-local weights informed by housing data or a satellite image.

## 1. Introduction

In real-world applications, satellite images, housing data, census data and other types of geographical data become highly relevant for modelling the probability of a certain type of event. The methodology presented here provides us a general framework paired with fast algorithms for incorporating external information in density estimation computations.

In density estimation, one is given a discrete sample of event locations, drawn from some unknown density *u* on the spatial domain, and tries to approximately recover *u* [1]. Relating the events to the additional data allows one to search over a smaller space of densities, which can yield more accurate results with fewer events. We refer to the additional data source as the function *g*(*x*) defined over the spatial domain *Ω*. We may typically assume two things about the relationship between *g* and *u*: (i) *g* informs the support of *u* via *g*(*x*)=0⇒*u*(*x*)=0 and (ii) *u* varies smoothly with *g* in a non-local way (explained below). This method allows the additional information in *g* to significantly improve the recovery of *u*.

### (a) Maximum penalized likelihood estimation

Although there are other classes of methods in the density estimation literature that are quite popular (such as average shifted histogram and kernel density estimation [2]), in this work we shall focus on maximum penalized likelihood estimation (MPLE). MPLE provides a general framework for finding an approximate density from sampled events. The likelihood of events occurring at the locations 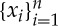 according to a proposed probability *u* is the product of the probability evaluated at each of those locations:

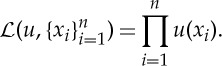

MPLE approximates *u* as the maximizer of a log-likelihood term combined with a penalty term, typically enforcing smoothness [3],

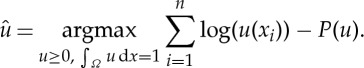

Without some kind of penalty term, the solution is just a weighted sum of Dirac deltas located at the training samples. Typical choices of *P*(*u*) include the total variation (TV) norm, 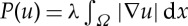, and the *H*^1^ Sobolev seminorm, 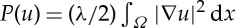. Here λ is the parameter that controls the amount of regularization. This is typically chosen via cross-validation, when it is computationally feasible.

### (b) Maximum penalized likelihood estimation applied to crime

The *H*^1^ seminorm is a common, well-understood regularizer in image processing related to Poisson's equation, the heat equation and the Weiner filter, producing visually smooth surfaces. For this reason, it is often a default choice when little is known about the data being modelled. *H*^1^ MPLE has further justification in crime density estimation from the ‘broken window’ effect [4–6]. This observation states that, after a burglary has occurred at a given house, burglaries are more likely to occur at the same house or nearby houses for some period of time afterwards. Initial burglaries give criminals information about what valuables remain and the schedule of inhabitants in the area. Additionally, a successful burglary leaves environmental clues, such as broken windows, that indicate that an area is more crime-tolerant than others. This effect leads to repeat and near-repeat burglaries. More generally, criminals tend to move in a bounded region around a few key nodes and have limited awareness of potential for criminal activity outside of familiar areas [7–9]. Within neighbourhoods, risk factors are typically homogeneous [10–12]. All of this explains why observed incidence rates of burglaries are locally smooth.

However, local smoothness is not always appropriate, and in practice there is much room for improvement. In recent years, several studies on the application of MPLE to crime data [13–15] emphasize the fact that crime density should have boundaries corresponding to the local geography. Mohler *et al.* [13] and Kostić *et al.* [15] model this by choosing penalty functions that are edge-preserving, TV and Ginzburg–Landau, respectively. Smith *et al.* [14] more closely follow the idea presented here. That work introduces a modified *H*^1^ MPLE, which based the penalty term on an additional component of the data. The method assumes that the valid region of the probability density estimate is known *a priori*. In their application to residential burglary, the valid region was the approximate support of the housing density in the region. If we denote the valid region by *D*, then the modified penalty term is just a standard *H*^1^ MPLE with a factor 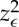 in the integral, where *z*_*ϵ*_ is a smooth Ambrosio–Tortorelli approximation of (1−*δ*(∂*D*)):

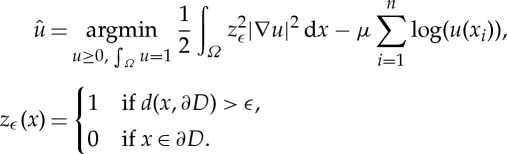



### (c) Graph-based methods

In spectral graph theory, data are represented as nodes of a weighted graph, where the weight on each edge indicates the similarity between the two nodes. Such data structures have been very successfully applied to data clustering problems and image segmentation [16–18]. The standard theory behind this is described in [19,20] and a tutorial on spectral clustering is given in [21]. A theory of non-local calculus was developed first by Zhou & Schölkopf in 2004 [22] and put in a continuous setting by Gilboa & Osher in 2008 [23]. Such methods were originally used for image denoising [23,24], but the general framework led to methods for inpainting, reconstruction and deblurring [25–29]. Compared with local methods, non-local methods are generally better able to handle images with patterns and texture. Further, by choosing an appropriate affinity function, the methods can be made suitable for a wide variety of different datasets, not just images.

In this article, we present non-local (NL) *H*^1^ MPLE, which modifies the standard *H*^1^ MPLE energy to account for spatial inhomogeneities, but unlike Smith *et al.* [14], we do so in a non-local way, which has the benefit of leveraging recent fast algorithms and the potential to generalize to other applications.

The organization of this article is as follows. In §2, we introduce the NL *H*^1^ MPLE method and review the non-local calculus and numerical methods on which it is based. In §3, we demonstrate the advantages of NL *H*^1^ MPLE by comparing it with standard *H*^1^ MPLE when applied to modelling residential burglary. In §4, we summarize our conclusions and discuss directions for future research.

## 2. Non-local crime density estimation

We propose replacing the *H*^1^ seminorm regularizer of *H*^1^ MPLE with a linear combination of an *H*^1^ regularizer and a non-local smoothing term 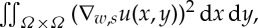 where ∇_*w*,*s*_ denotes the non-local symmetric-normalized gradient depending on an affinity function *w* derived from the spatial data, *g*. More details are found in §2*b*. The energy we optimize is thus
2.1
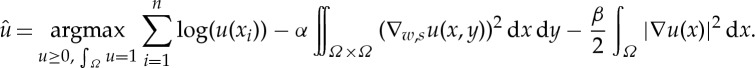



The non-local term in equation (2.1) is tolerant of sharp changes in the probability density estimate, as long as they coincide with sharp non-local changes in the spatial data. The mathematical formulation of this statement follows from the definitions presented in the following sections and is presented in appendix A. Before reviewing the non-local calculus behind this energy, we motivate why a non-local regularizer is good for crime density estimation. Many cities grow in a dispersal colony-like fashion, i.e. colony patches start growing at dispersed locations at the same time with the same architectural or cultural model as a starting point, generating non-local similarities [30]. Dissimilar colony patches grow and meet to form diffuse interface-like boundaries [31]. Thus, housing data typically contain similar features spread across the domain, along with interfaces between different types of areas. Whereas opposite sides of these interfaces are spatially close, they are non-locally well separated.

The clearest advantage of non-local regularization is that it allows for sharp changes in crime density across interfaces of distinct housing regions. In particular, because the residential areas are non-locally well separated from the non-residential areas, the non-local regularized estimate correctly captures the support of the residential burglary density. This feature has been studied for its own sake in prior work, and non-local regularization addresses it in an automatic, hands-off way.

Another, more subtle advantage of non-local regularization is that it encourages distant, but non-locally similar regions (e.g. colony patches based on the same model) to have similar crime density values. The assumption is that the layout of a neighbourhood and its crime density are both tied to underlying socio-economic factors. When one has these relevant factors, one can perform risk terrain modelling [12], combining the factors in the way that is most consistent with the observed data. Non-local regularization implicitly measures correlation between housing features and levels of crime, presumably explained by these unknown factors. The regularization encourages those relationships to remain consistent across the entire domain and all data. In this work, we base the non-local similarity of two locations on the similarity of surrounding housing density patches. For the sake of simplicity, one could consider basing it on only the housing density in the immediate vicinity. This would encourage the crime density to be a smooth function of the immediate housing density. Probably, one would estimate residential burglaries as roughly proportional to the housing density. This would be a simple, but reasonable null model, assuming that burglary depends heavily on opportunity. One would balance the spatial smoothness and smoothness as a function of housing density with cross-validation, allowing for varying results depending on what the data show. Our non-local weights are based on housing density patches, which makes them more noise-robust and representative of more complex housing features. This approach is general, relates to previous work in image processing and produces favourable results.

### (a) Non-local means

The technique of non-local means was originally developed for the application of image denoising, but can also be interpreted as an affinity function. The formula for the non-local means affinity, *w*_**Im**_, is given by [24]
2.2
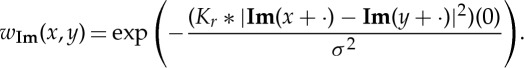

Here, **Im** is the image on which the non-local means weights are based, *K*_*r*_ is a non-negative weight kernel of size (2*r*+1)×(2*r*+1) and *σ* is a scaling parameter. This function measures similarity between two pixels based on a weighted ℓ^2^ difference between patches surrounding them in the image. In our experiments, the image **Im** is either a housing image or a satellite image. In practical settings, computing and storing all function values of *w* is a very computationally intensive task, so we use the fast approximation: Nyström's extension (see §2*d*).

### (b) Non-local calculus and graphs

Non-local calculus was introduced in its discrete form by Zhou & Schölkopf [22] and put in a continuous framework by Gilboa & Osher [23]. In these definitions, *w*(*x*,*y*) is a general non-negative symmetric affinity function, which generally measures similarity between the points *x* and *y*.

Let 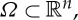 and *u*(*x*) be a function 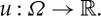 Then, the non-local gradient of *u* at the point *x*∈*Ω* in the direction of *y*∈*Ω* is given by

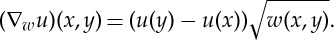

This suggests an analogous generalization of divergence, which in turn leads to the following definition of the non-local Laplacian:
2.3
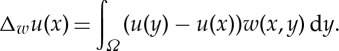



Now, let 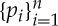 be a discrete subset of *Ω* and let *w*_*ij*_=*w*(*p*_*i*_,*p*_*j*_) if *i*≠*j* and *w*_*ii*_=0. We then let 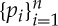 be vertices and *w*_*ij*_ the edge weights on a weighted graph. Let 
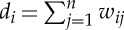
 be the weighted degree of the *i*th node. Then, the graph Laplacian applied to the function on the graph, *u*, is given by *Lu* where

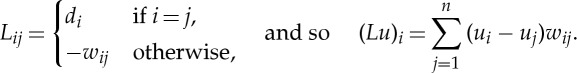

To keep the spectrum of the graph Laplacian in a fixed range as the number of samples in increased, and thus to guarantee consistency, we must normalize the graph Laplacian. See Bertozzi & Flenner [32] for a more in-depth discussion of this. We use the symmetric normalization.

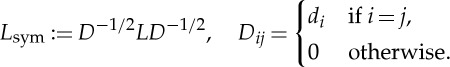

Because we express our energy as applied to functions over continuous domains, we also introduce the following notation for the symmetric-normalized non-local gradient:

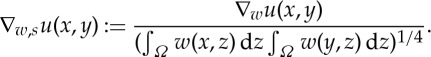



### (c) Numerical optimization

We must numerically find an approximate solution. The unconstrained energy has gradient flow

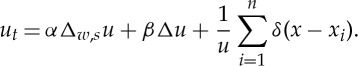

We evolve this equation, projecting onto the space of probability densities after each step. We discretize the equation as

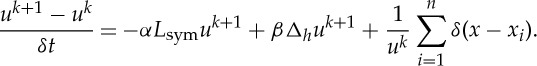

Here, Δ_*h*_ denotes the discrete Laplacian from the five-point finite difference stencil with mesh size *h*=1. Solving for *u*^*k*+1^ yields

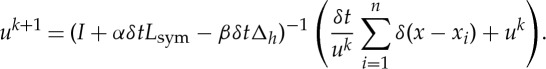

To approximate this, we use a split-time method:

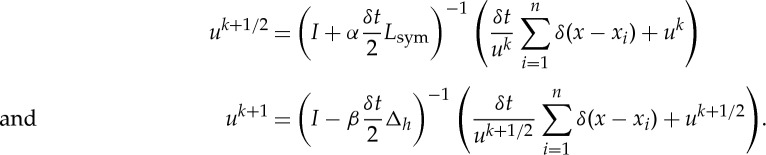

To apply these operators, we use a spectral method. This has two advantages over forming and multiplying the matrices. First, we can approximate the projection onto the constraint by using the spectral decomposition of the discrete Laplacian (shown in table 1). Second, the computation required to form and apply the entire symmetric graph Laplacian is also intensive. Fortunately, we can apply Nyström's extension (discussed in §2*d*), which is a popular method for approximating a portion of the eigenvectors and eigenvalues that approximate the operator well. To project onto the eigenvectors of Δ_*h*_, we apply the two-dimensional fast Fourier transform.

**Table 1.** Non-local *H*^1^ MPLE algorithm.

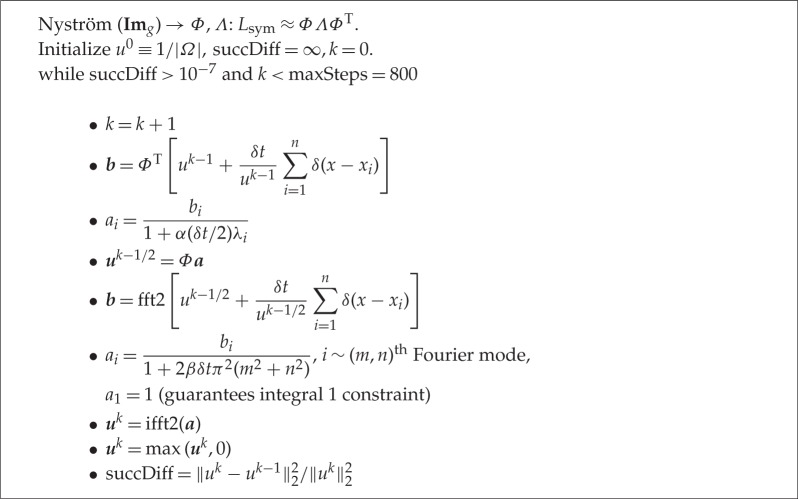

In both the case of applying (*I*+*α*(*δt*/2)*L*_sym_)^−1^ and (*I*−*β*(*δt*/2)Δ_*h*_)^−1^, we are applying operators of the form (*I*+*δtP*)^−1^, where *P* is symmetric and positive semidefinite. In general, if *P* has spectral decomposition *P*=*ΦΛΦ*^T^, then we apply (*I*+*δtP*)^−1^ to ***w*** by first projecting onto the eigenvectors, ***a***=*Φ*^T^***w***, updating the coefficients 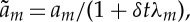 and finally transforming back to the standard basis, 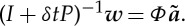 We summarize the steps of our algorithm in table 1.

### (d) Nyström's extension

To apply the spectral method described in the previous section, we need to approximate the eigenvectors and eigenvalues of the symmetric graph Laplacian. Here, we present Nyström's extension method and refer the reader to [25,32,33] for further discussion and analysis. Nyström's extension is a technique for performing matrix completion, well known within the spectral graph theory community. In this setting, Nyström's extension is applied to the normalized affinity matrix *W*_sym_=*D*^−1/2^*WD*^−1/2^, where the (*i*,*j*)th entry of *W* is the affinity between nodes *i* and *j*. Note that the matrices *W*_sym_ and *L*_sym_ have the same eigenvectors, and λ is an eigenvalue of *W*_sym_ if and only if 1−λ is an eigenvalue of *L*_sym_.

We let *N* denote the set of nodes in our complete weighted graph, then take *X* to be a small random sample from *N*, and *Y* its complement. Up to a permutation of the nodes, we can write the affinity matrix as

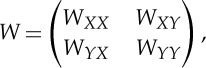

where the matrix 
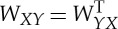
 consists of weights between nodes in *X* and nodes in *Y* , *W*_*XX*_ consists of weights between pairs of nodes in *X*, and *W*_*YY*_ consists of weights between pairs of nodes in *Y* . Nyström's extension approximates the eigenvalues and eigenvectors of the affinity matrix by manipulating the approximation:

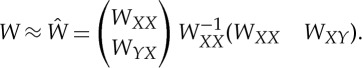

This approximates 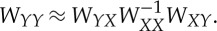 The error due to this approximation is determined by how well the rows of *W*_*XY*_ span the rows of *W*_*YY*_. If the affinity matrix *W* is positive semidefinite, then we can write it as a matrix transpose times itself: *W*=*V* ^T^*V* . In [34], the authors show that the Nyström extension thus approximates the unknown part of *V* (corresponding to *W*_*YY*_) by orthogonally projecting it onto the range of the known part (corresponding to *W*_*XY*_). In this setting, it is clear that, as the size of *X* grows, the approximation improves. Further, a random choice of *X* is likely to yield *W*_*XY*_ full rank if the rank of *W* is sufficiently large.

Next, we must incorporate the normalization factors into the above approximation. The degrees are approximated by applying their definition to the approximation. Note that 
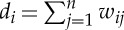
 can also be written as *d*=*W***1**_*n*_, where **1**_*n*_ is the length-*n* vector of ones. This yields

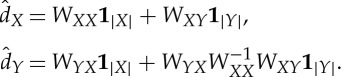

In this way, we approximate the degrees without forming any matrices of size larger than |*X*|×|*Y* |. Define also the vectors 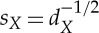 and 
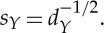
 Normalizing our approximation of *W* gives

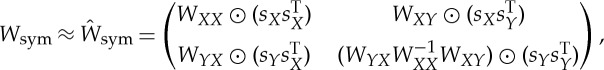

where 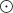 denotes component-wise product. For notational convenience going forward, let us define 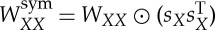 and 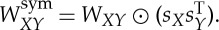


In practice, one uses a diagonal decomposition of such a formula to avoid forming and applying the full matrix. It follows from the analysis discussed in [33] that, if 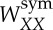 is positive definite, the diagonal decomposition of the approximation is given by 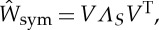 where

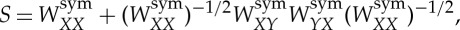

*S* has diagonal decomposition 
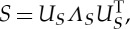
 and

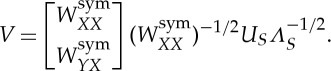



Note that *S* is size |*X*|×|*X*| and *V* is size |*N*|×|*X*|. Their computation never requires computing or storing matrices larger than size |*N*|×|*X*|. Thus, *V* is a matrix of |*X*| approximate eigenvectors of *W*_sym_ with corresponding eigenvalues *Λ*_*S*_. For more detailed discussion on Nyström's extension, see [25,32,33].

### (e) Cross-validation

Cross-validation is a methodology for choosing the smoothing parameter λ which yields probability densities that are predictive of the missing data [35]. Because our method consists primarily of simple coefficient updates after mapping to different eigenspaces, it is fast relative to methods with similar goals [14]. This speed increase allows us to perform 10-fold cross-validation, which requires many evaluations of the density estimation method. In *V* -fold cross-validation, we randomly partition the data points into *V* disjoint subsets 
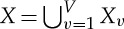
 with complements *X*_−*v*_=*X*∖*X*_*v*_. We let *u*_λ,−*v*_ denote the density estimate using parameter λ trained on the data *X*_−*v*_. The objective we minimize is an application of the Kullback–Leibler divergence, an asymmetric distance measure for probabilities given by

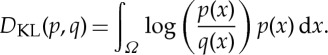

We select the parameter λ that minimizes the average KL divergence between the density estimates, *u*_λ,−*v*_, and the discrete distributions on the withheld data points,

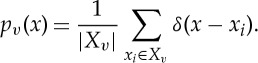

This yields the following optimization:

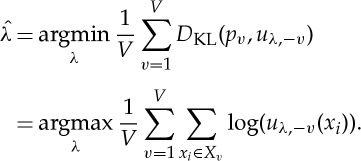

The result can also be interpreted as maximizing the average log-likelihood that the missing events are drawn from the corresponding estimated densities. We approximate this optimization via a grid search (note that λ=(*α*,*β*) is two-dimensional). The search requires the computation of all the density estimates *u*_λ,−*v*_. In particular, for 10-fold cross-validation, we must compute 10×|*α* values|×|*β* values| densities.

When evaluating the energy, it is important to ensure that non-negativity and sum-to-unity constraints hold strictly for the input densities. If a density is slightly negative somewhere, it could add complex terms to the objective, and if a density has sum slightly larger than unity, it could unfairly achieve a slightly higher objective. Further, in the strictest interpretation, if a density has a value zero at the location of a missing event, the objective will take value 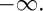 We relax this penalty by replacing *u*_λ,−*v*_(*x*_*i*_) with 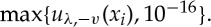


## 3. Numerical experiments

Here, we demonstrate the advantage of the NL *H*^1^ MPLE method over standard *H*^1^ MPLE by evaluating its performance on residential burglary data from San Fernando Valley, Los Angeles, CA, using corresponding housing data and a satellite image to inform the non-local weights.

### (a) Residential burglary

**Figure 1.**
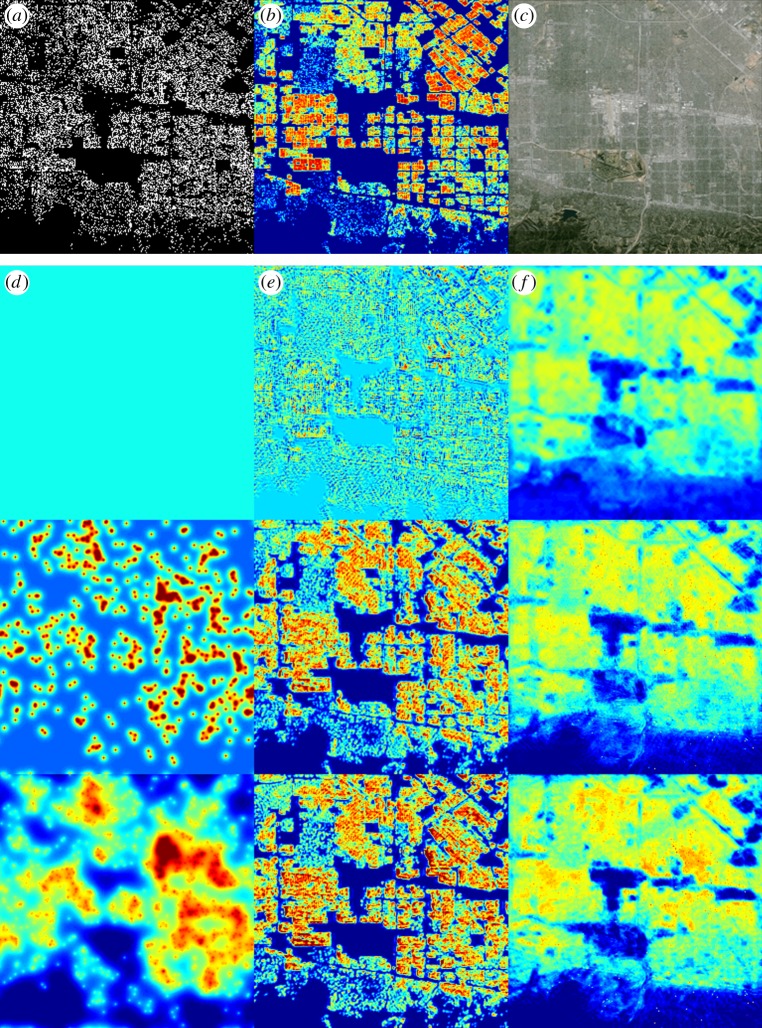
Top row: data. (*a*) 2005–2013 residential burglaries in San Fernando Valley (from LAPD). (*b*) San Fernando Valley 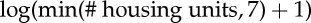 (from LA County Tax Assessor). (*c*) Satellite image of San Fernando Valley (from Google Maps). Bottom three rows: MPLE of 50, 500 and 1000 random samples from 2008 residential burglaries. (*d*) Column 1: *H*^1^ MPLE. (*e*) Column 2: housing NL *H*^1^ MPLE. (*f*) Column 3: satellite NL *H*^1^ MPLE. (Online version in colour.)

We perform experiments on residential burglary data from San Fernando Valley in 2005–2013, getting substantially different results from those shown in [13–15]. In figure 1, we show the data used (locations of residential burglaries in figure 1*a*, housing in figure 1*b* and satellite image in figure 1*c*), and *H*^1^ MPLE (figure 1*d*), housing-based NL *H*^1^ MPLE (figure 1*e*) and satellite-based NL *H*^1^ MPLE (figure 1*f*) density estimates on increasing subsets of data from 2005 to 2008. To evaluate performance, we compute the log-likelihood of each density on the residential burglaries from 2009 to 2013 (shown in table 2).

**Table 2.** Log-likelihood of densities on residential burglaries from 2009 to 2013 (corrected and raw). The maximum value in each row is shown in *italics*.

training dataset (corrected)	scaled histogram	*H*^1^	housing NL *H*^1^
50 random from 2008	−3.6039×10^5^	−*1.3386*×10^5^	−1.3396×10^5^
100 random from 2008	−3.5991×10^5^	−*1.3369*×10^5^	−1.3369×10^5^
500 random from 2008	−3.5197×10^5^	−1.3282×10^5^	−*1.3004*×10^5^
1000 random from 2008	−3.4350×10^5^	−1.3246×10^5^	−*1.2953*×10^5^
2008	−3.1905×10^5^	−1.3189×10^5^	−*1.2888*×10^5^
2007–2008	−2.9846×10^5^	−1.3174×10^5^	−*1.2850*×10^5^
2006–2008	−2.8152×10^5^	−1.3136×10^5^	−*1.2815*×10^5^
2005–2008	−2.6847×10^5^	−1.3121×10^5^	−*1.2774*×10^5^

training dataset (raw)	scaled histogram	*H*^1^	satellite NL *H*^1^
50 random from 2008	−3.6959×10^5^	−1.3733×10^5^	−*1.3553*×10^5^
100 random from 2008	−3.6822×10^5^	−1.3732×10^5^	−*1.3553*×10^5^
500 random from 2008	−3.6342×10^5^	−1.3583×10^5^	−*1.3524*×10^5^
1000 random from 2008	−3.5733×10^5^	−1.3598×10^5^	−*1.3525*×10^5^
2008	−3.3313×10^5^	−1.3535×10^5^	−*1.3494*×10^5^
2007–2008	−3.1326×10^5^	−1.3525×10^5^	−*1.3482*×10^5^
2006–2008	−2.9630×10^5^	−1.3496×10^5^	−*1.3449*×10^5^
2005–2008	−2.8334×10^5^	−1.3488×10^5^	−*1.3431*×10^5^

As one would predict, the locations of residential burglaries in figure 1*a* are primarily restricted to the support of the housing density image in figure 1*b*. There are some locations in the burglary dataset that correspond to locations with no residences (4173 events out of 23 725 total), which we attribute to imprecision in the burglary data. Most such misplaced events occur on streets, suggesting that the actual event took place at a residence facing that street. Because of this inconsistency between the datasets, for the experiments that use the housing data, we adjust the residential burglary data for training and testing (for both *H*^1^ and NL *H*^1^), moving each event to the nearest house if it is within two pixels, and dropping the event otherwise. This results in 603 dropped events. For the experiments that do not use housing data, we work with the raw burglary data for training and testing.

We implement *H*^1^ MPLE by applying our algorithm, described in table 1, with *α*=0 and *Φ*=Id. We choose the value of the regularization parameter *β* for each training dataset by performing 10-fold log-likelihood cross-validation, searching over *β*= [ 0,10. ^(-2:8)]. We apply *H*^1^ MPLE to both the raw and corrected burglary data.

For housing-based NL *H*^1^ MPLE, we perform Nyström's extension with non-local means applied to *g*, the housing density image shown in figure 1*b*. We use 400 random samples for Nyström's extension. We use the first 300 eigenvectors and eigenvalues in our computations. The non-local means weights are based on differences between patches of size 11×11 and *σ*=1⋅std(*g*), the standard deviation of the housing image. The weight kernel *K*_*r*_, *r*=5, is given as follows:

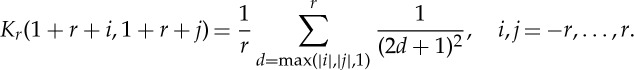

To choose the regularization parameters *α* and *β*, we perform 10-fold log-likelihood cross-validation, searching over *α*=[0,10.^(−2:12)] and *β*=[0,10.^(−2:8)]. We apply housing NL *H*^1^ MPLE to the corrected burglary data.

For satellite-based NL *H*^1^ MPLE, we perform Nyström's extension with non-local means applied to *g*, the Google Maps image shown in figure 1*c*. In applying non-local means to a colour image, we interpret the image as a vector-valued function with three components (one for each colour channel) and so, in equation (2.2), the expression |**Im**(*x*+⋅)−**Im**(*y*+⋅)|^2^ has size (2*r*+1)×(2*r*+1)×3. We use 800 random samples for Nyström's extension. We use the first 600 eigenvectors and eigenvalues in our computations. The non-local means weights are based on differences between patches of size 11×11 and *σ*=1⋅std(*g*), the standard deviation of the Google Maps image. The weight kernel is as in the previous case, but repeated on each colour channel. To choose the regularization parameters *α* and *β* for each training set, we perform 10-fold log-likelihood cross-validation, searching over *α*=[0,10.^(−2:12)] and *β*=[0,10.^(−2:8)]. We apply satellite NL *H*^1^ MPLE to the raw burglary data.

The *H*^1^ MPLE results transition from a completely smooth uniform density to a probability density with more apparent structure as the amount of training data increases. The NL *H*^1^ MPLE housing and satellite results exhibit a similar trend, but are able to better approximate the correct support of the density with many fewer data points. The measurable benefit of non-local smoothing is shown by the log-likelihood values in table 2. NL *H*^1^ generally gets higher log-likelihood than *H*^1^. This means that the densities estimated by housing NL *H*^1^ on corrected 2005–2008 data are more congruous with the corrected 2009–2013 data than the *H*^1^ densities, and the densities estimated by satellite NL *H*^1^ on raw 2005–2008 data are more congruous with the raw 2009–2013 data than the *H*^1^ densities.

The added complexity of our algorithm results in an increase in run time from the standard *H*^1^ MPLE, but the difference is not too substantial. We compare run times on a laptop with one Intel core i7 processor that has two cores with processor speed 2.67 GHz and 4 GB of memory. The run time for Nyström applied to the housing image is typically about 17 s. The run time for Nyström applied to the satellite image is typically about 36 s. For cross-validation purposes, Nyström can be run once outside of the loop and the results used for all combinations of datasets and parameters. The run time for *H*^1^ MPLE with parameters as chosen by cross-validation on the residential burglaries from 2005 to 2008 is typically about half a second. The run time for housing NL *H*^1^ MPLE with parameters as chosen by cross-validation on the residential burglaries from 2005 to 2008 is typically about 2.3 s. The run time for satellite NL *H*^1^ MPLE with parameters as chosen by cross-validation on the residential burglaries from 2005 to 2008 is typically about 1.5 s. The cross-validation run times depend on what range of parameters are being tested, but can easily be run in parallel across several computing nodes.

### (b) Synthetic density

**Figure 2.**
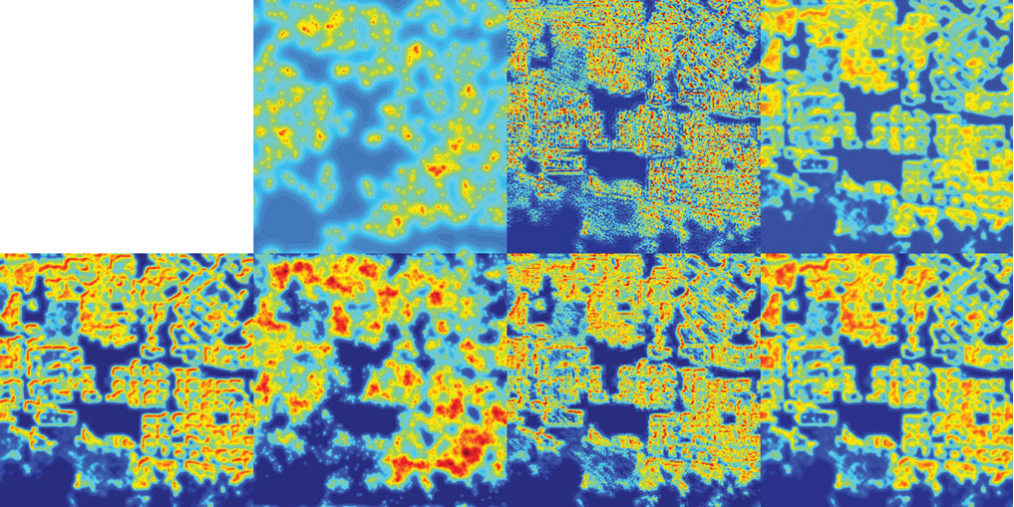
Synthetic density recovery (see §3*b*). Top row: density estimates based on 400 samples from synthetic density. 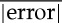: *H*^1^ 7.12473×10^−6^, NL *H*^1^ 5.26617×10^−6^ , NL *H*^1^ restricted 2.55042×10^−6^. Bottom row: synthetic density and density estimates on 4000 samples. 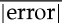: *H*^1^ 5.05662×10^−6^, NL *H*^1^ 2.52831×10^−6^ , NL *H*^1^ restricted 1.36416×10^−6^. (Online version in colour.)

To further verify that NL *H*^1^ MPLE is correctly performing density estimation, we test the method's ability to recover a given density. We start with a known density, draw events from it and attempt to recover it. Because the method assumes a relationship between the spatial data *g* and the density *u*, we generate a synthetic density that is closely related to the housing data, shown in the bottom left of figure 2. This density is given by taking a random linear combination of the first five approximated eigenvectors of the graph Laplacian (with weights based on the housing image) and then shifting and normalizing the result to yield a probability density. The coefficients are chosen uniformly at random in [0,1] and the non-local weights are based on the housing data as they were in the previous section. This randomly generated density was chosen over others because it looks like a potential probability density for residential burglary. It should be noted that this choice of synthetic density is quite ideal for the proposed method. The hope is that very good density recovery of ideal probability densities extends to good density recovery of less ideal probability densities.

We sample the events according to this density by generating numbers uniformly at random in [0,1] and inverting the cumulative distribution function associated with the density. In the top row of figure 2, we show the *H*^1^ MPLE result on the 400 events (*β*=5×10^4^), the housing NL *H*^1^ MPLE result on the 400 events (*α*=100,*β*=0) and the NL *H*^1^ MPLE result on 400 events restricted to the first five eigenvectors. In the bottom row of figure 2, we show the synthetic density, the *H*^1^ MPLE result on the 4000 events (*β*=10^5^), the housing NL *H*^1^ MPLE result on the 4000 events (*α*=10^8^,*β*=0) and the NL *H*^1^ MPLE result on 4000 events restricted to the first five eigenvectors. In all the cases, smoothing parameters were chosen to minimize the mean absolute error of the probability density. The NL *H*^1^ results and the restricted NL *H*^1^ results do a substantially better job at recovering the probability density than *H*^1^ MPLE. This is expected of course, from the construction of the probability. The comparison merely suggests that, if the correct density is well approximated by a combination of eigenvectors of the graph Laplacian, enforcing non-local smoothness can substantially improve recovery of the density. It is, in general, difficult to determine when a density is well approximated by a graph Laplacian's eigenbasis. The assumption is that the primary and non-local data have some meaningful, consistent connection. We refer the reader to §2 for heuristics on this connection and the appendix for some more precise formulations. It is also worth noting that, if unrelated non-local data are used, cross-validation will probably yield *α*=0, reverting the model back to standard *H*^1^ MPLE.

## 4. Conclusion and future work

In this paper, we have looked at the problem of obtaining spatially accurate probability density estimates. The need for new approaches is demonstrated by the inadequate performance of standard techniques such as *H*^1^ MPLE.

Our proposed solution accomplishes this by incorporating a non-local regularity term based on the *H*^1^ regularizer and non-local means, which fuses geographical information into the density estimate. Our experiments with the San Fernando Valley residential burglary dataset demonstrate that this method does yield a probability density estimate with the correct support that also gives favourable log-likelihood results. Further, our results based on the Google Maps image suggest that we can apply NL *H*^1^ MPLE to a wide variety of geographical regions without obtaining specialized geographical data.

There are several other aspects of this and related problems to explore. In general, testing the method on other datasets would be interesting. This may present the added challenge of dealing with other types of geographical information, because high-resolution housing density data may not be readily available. In modelling the density of other types of events, the geographical data may not be related to housing at all. As the problem dictates, the non-local weights can be replaced with whatever weights seem appropriate for the data at hand. We have yet to incorporate time, leading indicators of crime, or census data into the model. Any of these could further improve results and allow one to use density estimation in place of risk terrain modelling.

Finally, our method need not stand alone. Several sophisticated spatio-temporal models for probabilistic events make use of density estimation, typically using the standard methods [36–38]. By replacing the standard density estimation techniques with a non-locally regularized MPLE such as ours, the density estimates in these models could improve, thus improving the overall result of the resulting simulation.

## Supplementary Material

readme.txt

## Supplementary Material

NLH1MPLE_SynthDensScript.m

## Supplementary Material

NLH1MPLE.m

## Supplementary Material

NystromEigsSeedBS.m

## Supplementary Material

SFHouses.csv

## Supplementary Material

SFSat.csv

## Supplementary Material

SynthDens.csv

## Data accessibility

The crime data cannot be shared because it contains human subject data. The housing data, satellite image and synthetic density are all available in the electronic supplementary material.

## Appendix A

To examine the effect of the non-local regularization term, we compute an alternative formulation of the NL *H*^1^ MPLE problem and derive an inequality that solutions must satisfy. Recall from equation (2.1) that NL *H*^1^ MPLE applied to the event samples  with parameters *α*,*β*≥0 is given by the following optimization:

For every such *X*,*α*,*β*, one can show that there exist non-negative constants *C*_1_ and *C*_2_ such that *u*_*α*,*β*,*X*_ is also the solution to a more constrained optimization:

A 1

It can further be shown that, for *X* and *β*≥0 fixed, *C*_1_ is a non-increasing function of *α*≥0 and, for *X* and *α*≥0 fixed, *C*_2_ is a non-increasing function of *β*≥0.

Any solution of equation (.1) satisfies  and likewise in the discrete setting we have the following:

Thus, for some non-negative discrete function  with  we have the following:

A 2

Recalling that, in our application, we set the weights *w*_*ij*_ to be non-local means applied to a housing image,  we can interpret what this means. Up to some factors constrained by the parameter *C*_1_, the squared difference between the density at pixels *i* and *j* is bounded by  Thus, the bound is made restrictive when: *d*_*i*_ and *d*_*j*_ are small, which means the patches of *g* around pixels *i* and *j* are very different from the rest of the image; and when *w*_*ij*_ is large, which means the neighbourhoods of *g* around pixels *i* and *j* are similar to each other.

It is also worth noting that, by constraint, the left-hand side of (.2) is always smaller than or equal to 1. Thus, for the inequality to be non-trivial, we must have  for some pair *i*,*j*∈*Ω*. Thus, *C*_1_ must be sufficiently small (or *α* sufficiently large) in order to guarantee that the non-local smoothing will have any effect on *u*.
